# Care practices and traditional beliefs related to neonatal jaundice in northern Vietnam: a population-based, cross-sectional descriptive study

**DOI:** 10.1186/1471-2431-14-264

**Published:** 2014-10-14

**Authors:** Loc T Le, John Colin Partridge, Bich H Tran, Vui T Le, Tuan K Duong, Ha T Nguyen, Thomas B Newman

**Affiliations:** Department of Pediatrics, University of California, San Francisco, Box 0748, 533 Parnassus Ave, U585, San Francisco, CA 94143 USA; CHILILAB, Hanoi School of Public Health, 138 Giang Vo, Hanoi, Vietnam; Department of Epidemiology & Biostatistics, University of California, San Francisco, Box 0560, 185 Berry Street W, Suite 5700, San Francisco, CA 94143 USA

**Keywords:** Hyperbilirubinemia, Newborn, Care-seeking behavior, Vietnam, Traditional medicine, Phototherapy

## Abstract

**Background:**

The National Hospital of Pediatrics in Vietnam performed >200 exchange transfusions annually (2006–08), often on infants presenting encephalopathic from lower-level hospitals. As factors delaying care-seeking are not known, we sought to study care practices and traditional beliefs relating to neonatal jaundice in northern Vietnam.

**Methods:**

We conducted a prospective, cross-sectional, population-based, descriptive study from November 2008 through February 2010. We prospectively identified mothers of newborns through an on-going regional cohort study. Trained research assistants administered a 78-item questionnaire to mothers during home visits 14–28 days after birth except those we could not contact or whose babies remained hospitalized at 28 days.

**Results:**

We enrolled 979 mothers; 99% delivered at a health facility. Infants were discharged at a median age of 1.35 days. Only 11% received jaundice education; only 27% thought jaundice could be harmful. During the first week, 77% of newborns were kept in dark rooms. Only 2.5% had routine follow-up before 14 days. Among 118 mothers who were worried by their infant’s jaundice but did not seek care, 40% held non-medical beliefs about its cause or used traditional therapies instead of seeking care. Phototherapy was uncommon: 6 (0.6%) were treated before discharge and 3 (0.3%) on readmission. However, there were no exchange transfusions, kernicterus cases, or deaths.

**Conclusions:**

Early discharge without follow-up, low maternal knowledge, cultural practices, and use of traditional treatments may limit or delay detection or care-seeking for jaundice. However, in spite of the high prevalence of these practices and the low frequency of treatment, no bad outcomes were seen in this study of nearly 1,000 newborns.

**Electronic supplementary material:**

The online version of this article (doi:10.1186/1471-2431-14-264) contains supplementary material, which is available to authorized users.

## Background

Severe hyperbilirubinemia and kernicterus are rare in developed countries where bilirubin screening, blood typing, phototherapy equipment, and Rh immune globulin are available. In developing countries where these preventive therapeutic interventions are often unavailable, severe hyperbilirubinemia causes significant morbidity and mortality [1–14]. In Vietnam, the lack of blood type testing, Rh immune globulin and accessible phototherapy may in part explain the frequent use of exchange transfusion for the treatment of severe hyperbilirubinemia [15, 16].

At the National Hospital of Pediatrics (NHP), the tertiary referral hospital for > 31 million people in northern Vietnam, 18% of neonatal admissions in 2002 were for hyperbilirubinemia, 22% of babies admitted for jaundice from 2003–05 underwent exchange transfusion, and an average of 207 exchange transfusions were performed yearly in 2006–08 [15].

A case series we conducted of infants undergoing exchange transfusion at the NHP suggested that delays in diagnosis and treatment contributed significantly to the use of exchange transfusion to treat severe hyperbilirubinemia [16]. That study, however, provided no quantitative data about the barriers causing delays in care-seeking among those not receiving exchange transfusion, and hence could not quantify their importance as risk factors for severe hyperbilirubinemia. We speculated that low parental knowledge of jaundice, traditional non-medical beliefs about causes and treatment, wrong medical advice, and inefficient transport procedures likely contributed to delayed care-seeking in some infants. In this study, we describe the prevalence of community care practices and traditional beliefs that may contribute to delayed presentation with severe hyperbilirubinemia and the frequency of phototherapy use.

## Methods

We conducted a prospective, cross-sectional, descriptive population-based study at CHILILAB, a demographic and epidemiologic surveillance system established in 2003 by the Hanoi School of Public Health (HSPH) for public health and health policy research, from November 2008 through February 2010. CHILILAB is a member of the INDEPTH Network, an international network of field labs in 20 nations around the world that supports the development of longitudinal sites for health and social science research as well as intervention impact assessments. Located in Chi Linh District, Hai Duong Province (55 kilometers northeast of Hanoi), CHILILAB is comprised of 4 rural communes and 3 towns, with a study population of approximately 57,000 inhabitants from about 18,000 households. The entire district contains 17 communes and 3 towns, with a population of 142,278 (2010) [17].

The district public health care system consists of 1 district hospital, a regional health clinic, and 20 commune health stations. Commune health stations have nurse midwives who attend low risk vaginal deliveries while district hospitals have physicians who are able to perform C-sections. High risk deliveries are transferred to provincial or national hospitals. Sick neonates are usually transferred to the nearest neonatal intensive care unit at the provincial hospital 34 kilometers away or to the National Hospital of Pediatrics in Hanoi. Rapidly urbanizing and industrializing, Chi Linh District mirrors the socio-economic and demographic changes occurring throughout Vietnam [17].

We obtained research approval from both the HSPH Institutional Ethical Review Board (Approval #057/2008/YTCC-HD3) and University of California, San Francisco, Committee for Human Research (Approval #H63168-33205-01) in accordance with the Declaration of Helsinki. The study was approved locally by the Chi Linh District People’s Committee and district health officials.

We developed a questionnaire with input from Vietnamese physicians and public health faculty at the Hanoi School of Public Health to evaluate maternal knowledge of jaundice, to assess community newborn care practices that may affect jaundice detection and care-seeking behavior, and to determine the incidence of phototherapy. We then conducted a training session for the research assistants, reviewing the questionnaire and interview techniques before piloting the study for 2 weeks. Afterwards, we reconvened to address any problems and to revise the questionnaire with input from the research assistants, all of whom live in the community and have understanding of local care practices, before commencing the study.

We identified all expectant mothers through weekly telephone contact at commune health stations and the Chi Linh District Hospital where they were receiving prenatal care. All pregnant women are allowed a limited number of free prenatal care visits through the socialized government health care system which allows identification of pregnant mothers. Through prenatal and delivery records, we obtained their estimated delivery dates, and identified deliveries that occurred within the prior week. Mothers who were transferred to higher level hospitals due to complicated deliveries or electively delivered outside of the catchment area were captured during home visits conducted after their estimated date of delivery. The research assistants conducted home visits at 14–28 days after birth, and travelled on foot, bicycle, or motorcycle to reach the households. With these measures, we believe that we were able to identify nearly all and enroll most live births.

All consenting mothers of live-born infants in the CHILILAB surveillance area were included except those we could not contact or whose babies remained hospitalized at 28 days. After obtaining informed consent, the research assistants administered a 78-item questionnaire to the mother (Additional files 1 and 2). The questionnaire asked household demographic information, birth history, birth complications, presence of cephalohematoma, length of stay, newborn feeding, care practices, exposure or avoidance of sunlight, beliefs about effects of sunlight, use of traditional remedies, herbal medications, Chinese medicines, umbilical cord care, home environment, maternal knowledge of jaundice, maternal recognition and concern about jaundice, sibling history of jaundice, care seeking for jaundice, newborn follow-up care, symptoms of kernicterus, newborn re-hospitalization, phototherapy, and treatment history. We asked whether cost, distance, bad weather, poor perception of health providers, “baby was too young to take outside”, and lack of transportation were barriers to care. Mothers could choose more than one barrier and also could give an open response for other perceived barriers. Socio-economic data were extracted from the existing CHILILAB database, and class was categorized according to a standardized assessment of household wealth based upon possessions, home structure, and utilities.

We entered data into Microsoft Access, and then exported to STATA 11 (Statacorp, College Station, TX) for analysis. We used descriptive statistics, t-tests, chi-squared tests, and Wilcoxon rank sum tests to measure various associations with receiving phototherapy.

## Results

### Demographics

Based upon Chi Linh District’s crude birth rate of 15.6 per 1,000 people [17], we expected approximately 1,186 births in the CHILILAB’s catchment population of 57,000 over the 16 month study period. We identified 1,058 total births, of whom 61 (5.8%) were lost to follow-up, and 10 (0.9%) were excluded for hospitalization >28 days. Eight (0.8%) mothers declined participation, leaving 979 (93%) eligible infants with mothers consenting for participation. Over half resided in rural areas. Economic status of households was distributed evenly across economic quintiles. The most common head-of-household occupations were farming (28%), small business owner/trade worker (21%), factory worker/laborer (21%), and government official (10%). The vast majority (96%) of mothers had attended secondary (middle) school or higher. Illiteracy is low (0.5%) compared to the worst affected communes in CHILILAB (2-4%), [18] (Table 1) and compared to overall adult illiteracy in Vietnam (6.6%) (2008–12) [19].

**Table 1:** **Demographic & socio-economic status**

Demographic information, N = 979		
Maternal Age (years) (mean ± SD, range)	26.5 ± 4.9	(16, 46)
Gravity (mean ± SD, range)	1.8 ± 1.0	(1,6)
Parity (mean ± SD, range)	1.6 ± 0.7	(1, 6)
Rural residence (n,%)*	496	54%
Household economic Quintile, N = 913*	**N**	**%**
Lowest	161	18
Below average	208	23
Average	231	25
Above average	175	19
Highest	138	15
Total	913	100
Occupation of head of household, N = 781*	**n**	**%**
Farmer	222	28
Business owner/trade worker	164	21
Factory worker/laborer	163	21
Government official	81	10
Handicraft	62	8
In vocational training	53	7
Unemployed	17	2
Student	3	<1
Housewife	3	<1
Retired	2	<1
Elderly	1	<1
Other	10	1
Total	781	100
Education level, N = 781*	**n**	**%**
Illiterate	4	0.5
Primary school	27	3
Secondary school	378	48
High school	205	26
Vocational school	69	9
College	30	4
University	63	8
Post-graduate	5	0.6
Total	781	100

*Extracted from CHILILAB database. Data available on fewer subjects due to migration between periods of household assessment.

### Hospitalization and jaundice treatment

We obtained information on birth location, delivery history, hospitalization course, maternal and neonatal complications, and newborn readmissions within 14 days of delivery. Most (76%) delivered locally at either commune health stations or the district hospital, and <1% delivered at home. The median hospital stay after birth was 1.35 days. Almost 3% (n = 28) of infants had perinatal complications (by maternal report); the most common and medically significant were birth asphyxia, jaundice, respiratory distress/apnea, prematurity/small for gestational age, infection, and meconium/amniotic fluid aspiration. Four percent (n = 40) had birth weight <2,500 grams. Of the 924 mothers who knew the gestational age of their infants, 1% (n = 10) were 32–33 weeks gestation, and 14% (n = 130) were 34–36 weeks gestation. Because surfactant and adequate total peripheral nutrition were not yet routinely available at the National Hospital of Pediatrics at the time of this study, premature infants born <32 weeks gestation have variable survival, and those <28 weeks gestation typically do not survive due to limitations in neonatal care. Surviving infants <32 weeks typically would be hospitalized beyond 28 days, and would not be included in our sample. The readmission rate was 0.9% (n = 9) (Table 2), with 3 for pneumonia, 1 for prematurity, pneumonia and jaundice, 1 for brain hemorrhage, apnea and jaundice, 1 for gastrointestinal infection and jaundice, 1 for emesis, 1 for fever, and 1 for umbilical bleeding.

**Table 2:** **Birth history, complications & hospitalizations**

Birth location, N = 979	n	%
Commune health station	180	18
District hospital	561	57
Provincial hospital	189	19
Private clinic	6	<1
Home	9	<1
Other hospital	34	4
**Birth clinical history, N = 979**		
Cesarean section delivery (n,%)	157	16
Male newborn (n,%)	508	52
Birth weight (grams)(mean ± SD, range)	3132 ± 416	(1400, 5000)
Estimated Gestational Age (weeks) (mean ± SD, range)	38.9 ± 1.6	(32, 44)
Length of stay (median days)	1.35	-
Maternal complications during childbirth (n,%)	46	5
Infant complications during birth or birth hospitalization (n,%)	28	3
Cephalohematoma (n,%)	23	2
**Infant perinatal complications, N = 28** (excludes readmissions)	**n**	**%**
Asphyxia	4	14
Jaundice	3	11
Respiratory distress/apnea	3	11
Prematurity/small for gestational age	3	11
Eye discharge	3	11
Infection	2	7
Cephalohematoma	2	7
Separated sutures	2	7
Meconium/amniotic fluid aspiration	2	7
No urination	1	4
Poor feeding	1	4
Rash	1	4
Don’t know	1	4
**Hospitalization & treatments, N = 979**	**n**	**%**
Birth hospitalization for newborn illness	10	1
Readmissions	9	0.9
Phototherapy during birth hospitalization	6	0.6
Phototherapy during readmission	3	0.3
Phototherapy during birth hospitalization or with readmission	9	0.9
Exchange transfusion during birth hospitalization or readmission	0	0
Deaths	0	0

For infants who had an extended birth hospitalization or were readmitted, we asked if they received phototherapy or exchange transfusion and their outcome. Overall, 9 (0.9%) received phototherapy during the neonatal period. Six (0.6%) received phototherapy during the birth hospitalization. Three (0.3%) were readmitted for jaundice with co-morbidities of pneumonia, gastrointestinal infection, or apnea due to brain hemorrhage. There were no readmissions for isolated hyperbilirubinemia, no exchange transfusions, kernicterus cases, or deaths. All sick newborns were transferred to provincial or national hospitals for levels of care that could not be provided at the district hospital or commune health centers. We did not have access to bilirubin or other laboratory measurements for sick infants treated at hospitals outside of Chi Linh.

### Care practices, risk factors, and beliefs

The majority of women initially breast- and formula-fed their infants while waiting for their breast milk to come in. After the first week, however, the vast majority exclusively breast-fed. Exclusive formula feeding was rare. Mothball use, a possible trigger for hemolysis in newborns with G6PD deficiency, was reported by 43 mothers (4%). Families commonly avoided exposure of their newborns to direct sunlight (88%) and kept their newborns in dark rooms during the first 7 days (77%). When asked about the effects of sunlight on newborns, 33% (n = 320) thought sunlight was harmful of whom 171 thought it damaged the skin and/or eyes, 142 believed newborns were “too young/weak” and “would get sick” if exposed, and 2 thought it caused congestion (Table 3). We assessed maternal knowledge of jaundice and found that less than half had ever heard of newborn jaundice, and only 27% thought that jaundice could potentially be harmful. Only 11% received teaching on jaundice after birth (Table 4).

**Table 3:** **Feeding, home environment, beliefs, care practices, & barriers to care, N = 979**

Feedings during first 3 days	n	%
Exclusively breastfed	279	28
Breast and formula	671	69
Exclusively formula fed	26	3
Breast milk and rice water	2	<1
Breast milk and sugar water	1	<1
**Feedings after 7 days**		
Exclusively breast milk	751	77
Both breast milk & formula	223	23
Exclusively Formula Fed	5	<1
**Home environment**	**n**	**%**
Used mothballs in the home	43	4
Avoided newborn exposure to sunlight	864	88
Kept newborn in dark, enclosed room	750	77
**Beliefs of effects of sunlight**	**n**	**%**
Harmful	320	33
Beneficial	267	27
Neither harmful or beneficial	17	2
Don’t know	375	38
**Traditional newborn care practices**	**n**	**%**
Used traditional, herbal, or over-the counter medication during 1st week	164	17
Used herbs to treat jaundice	30	3
Scraped off white oral papules “lanh” to treat jaundice	1	0.1
**Perceived barriers to care**	**n**	**%**
Cost	171	17
Distance to care	77	8
Bad weather	76	8
Poor perception of health care providers	69	7
Baby too small/young to take outside	50	5
Lack of transportation	46	5

**Table 4:** **Care-seeking behavior & jaundice knowledge**

Follow-up newborn care, N = 979	n	%
Sought routine, well-baby care during first 2 weeks	24	2
Sought care for medical concerns during first 2 weeks	60	6
**Knowledge & recognition of jaundice, N = 979**	**n**	**%**
Had any knowledge of jaundice prior to delivery	439	45
Had knowledge that jaundice can be harmful	261	27
Received jaundice education from health providers after birth	105	11
Infant appeared jaundiced during first week	206	21
**Did you worry about the jaundice and seek care? N = 206**	**n**	**%**
Worried, sought medical care	22	11
Not worried, did not seek medical care	66	32
Worried, but did not seek medical care	118	57
**Reason for not seeking care for jaundice, N = 118**	**n**	**%**
Physiologic jaundice, self-limited	45	38
Caused by lanh	21	18
Caused by separation of sutures/fontanelle	20	17
Caused by both lanh and separation of sutures/fontanelle	1	<1
Still hospitalized	8	7
Not severe, monitored at home	6	5
Used herbal bath	2	2
Believed once skin exfoliates, jaundice will resolve	2	2
Weather too cold	2	2
Brought to traditional healer	1	<1
Placed infant in the sun	1	<1
Only recently became jaundiced	1	<1
Asked advice from a physician	1	<1
Was too busy	1	<1
Was advised by people not to worry	1	<1
Normal to have skin color changes	1	<1
Don’t know, no response	4	3

We inquired about the home use of traditional, herbal, or over-the-counter treatments, as these might delay care-seeking behavior and newborn follow-up care. One-hundred sixty-four (17%) mothers reported using traditional, herbal, or over-the-counter treatments for newborn problems during the first week. Thirty (3%) used herbal remedies to treat jaundice. Although cost was the most commonly reported potential barrier to care (17%), the majority reported none (Table 3). Routine well-baby follow-up care before 14 days was rare (2.5%); 6% of mothers sought newborn care for medical concerns during the first 2 weeks after birth (Table 4).

Of the 206 mothers who thought their infants appeared jaundiced after birth (“vang da” - translated literally as “yellow skin”), we asked if they were worried or sought care to assess their degree of concern and understand care-seeking behaviour. Thirty-two percent (n = 66) were not worried and did not seek care; 11% (n = 22) were worried and sought care; 57% (n = 118) were worried, but did not seek care. Of the 118 mothers who were worried but had not sought care, we inquired why they had not done so. Forty percent (n = 47) offered non-medical explanations for the cause of jaundice or treated it with traditional or herbal therapies at home. Responses of these 47 mothers included “separation/widening of skull sutures and fontanel”, “treated with herbal bath”, “brought to traditional healer”, “would resolve once skin peeled,” and “lanh”, white oral lesions that we suspect was thrush (Table 4).

We evaluated for associations between receiving phototherapy and various risk factors but could not make any conclusions due to limited power with only 9 infants receiving phototherapy.

## Discussion

This population-based, cross-sectional study of nearly 1,000 infants found a high rate of beliefs and practices that might put babies at risk for severe jaundice, but little evidence of severe hyperbilirubinemia or acute bilirubin encephalopathy in contrast to the high numbers of exchange transfusion performed at the NHP. Only 9 (0.9%) received phototherapy, 6 (0.6%) of which were during birth hospitalization, and 3 (0.3%) were readmissions. The readmission rate was lower than expected based on U.S. studies in which Asian newborns have higher risk [20–22] and where readmission rates for phototherapy for all races combined ranged between 0.45-3% [20, 21, 23–25]. Rates of readmission were further reduced in 2 studies to 0.43% and 0.18% after the implementation of routine bilirubin screening prior to discharge [24, 25]. Pre-discharge bilirubin screening and phototherapy, however, were not available in this community, and therefore, cannot explain the low rate of readmission for jaundice.

The absence of exchange transfusion or kernicterus was not surprising because our study was not designed nor powered to detect them. We conservatively estimated that there was 1 exchange transfusion per 2,545 births in northern Vietnam based upon an NHP catchment population of 31 million, crude national birth rate of 17.0 per 1,000 people (2007–09) [26], and average of 207 exchange transfusions performed yearly at the NHP (assuming exchange transfusions were performed exclusively at the NHP). However, we were expecting to find more cases of infants undergoing phototherapy and possibly detect cases of acute bilirubin encephalopathy but found none.

Selection and referral bias may have contributed to these unexpectedly low numbers. Our death rate was zero, and rate of birth complication was low, suggesting a relatively healthy newborn population compared to studies at the NHP. We excluded infants who remained hospitalized after 28 days, which meant premature infants <32 weeks gestation or other sick newborns with prolonged hospitalizations, who were at higher risk for hyperbilirubinemia, would have been excluded. A study of 615 newborns admitted for hyperbilirubinemia at the NHP (2003–05) found that 72% were low birth weight (<2500 g), 64% were premature (≤36 weeks), 10% had infection, 11% had birth asphyxia, and 17% had set-up for ABO-incompatibility [15]. Our case-series of 20 infants transferred to the NHP who underwent exchange transfusion (2008–2009) also showed that they were a high risk group. Seventy percent were transferred during birth hospitalization with most having co-morbidities of low birth weight, prematurity, infection, or Coombs + hemolysis. Half (n = 10) were delivered at tertiary centers (provincial or national hospitals) which selected for a higher risk population. Only 30% were readmissions from home [16]. Both selection and referral bias may explain the paradox of high numbers of exchange transfusion and acute bilirubin encephalopathy at the NHP but low rates of phototherapy and rarity of complications of hyperbilirubinemia in a population-based study.

Another explanation for low rates of phototherapy may be that hyperbilirubinemia went unnoticed, undetected, or untreated. Early discharge, lack of follow-up, and low parental knowledge of jaundice leading to decreased care-seeking may all contribute to lack of detection or treatment. Common cultural practices such as keeping infants in dark rooms during the first week after birth may also hinder jaundice detection at home, causing under-reporting. Because we used maternal reporting to estimate the incidence of jaundice without information from a medical assessment, clinical jaundice may have been under-estimated. Among mothers who reported their infant appeared jaundiced, many were not concerned, did not seek care, or used traditional therapies in lieu of care-seeking. It is possible that many infants became severely jaundiced but were not identified medically, and that we did not detect any bad outcomes because we did not have long-term follow-up which might have detected hearing loss or cerebral palsy, the long-term complications of untreated acute bilirubin encephalopathy. The lack of long-term follow-up is one of the main limitations of this study. Lastly, the common use of formula supplementation during the first week of life may have been protective [27, 28].

Concurrent, on-going efforts by the government and NGOs to implement phototherapy at provincial hospitals may have confounded our results by decreasing the incidence of hyperbilirubinemia and kernicterus during our study. The East Meets West Foundation started distributing LED phototherapy and supported courses on basic newborn care, including jaundice management, to 136 hospitals in Vietnam from 2007–09. Jaundice admissions to the NHP dropped yearly from 865 in 2008 to 509 in 2010. Kernicterus cases, however, remained unchanged between 2008 (n = 87) and 2010 (n = 81), and only dropped significantly in 2011 (n = 25, Jan-Sep) after the initiation of intensive jaundice workshops [29]. Although we cannot exclude this confounder, we believe it was unlikely that our low rates of phototherapy was an outcome of this intervention because the most dramatic changes in referrals for jaundice and kernicterus to the NHP occurred in 2011 after the conclusion of our data collection. Chi Linh District Hospital did not receive equipment or training during the study period. In addition, we would have expected increased utilization of phototherapy.

Although we were unable to determine the population incidence of hyperbilirubinemia, our study, nevertheless, contributes to understanding of perceptions of jaundice, care-seeking behavior for jaundice, and barriers to care. Our data could be used to develop educational interventions to dispel myths and improve care-seeking. Traditional, non-medical beliefs about the causes of jaundice and the use of traditional remedies in lieu of care-seeking were prevalent. Some mothers believed cranial suture diastases could cause jaundice and would treat the diastases and jaundice with an herb (typically burning the “ngai” herb and exposing the infant to the ashes or smoke). The association between jaundice and cranial suture diastasis may have arisen due to both being caused by intracranial hemorrhage which is frequent in Vietnam due to lack of vitamin K prophylaxis [30]. Others believed jaundice was caused by “lanh”. These lesions reportedly developed after the first few days of life and were believed to be associated with jaundice and poor feeding. Mothers said that jaundice would resolve and feeding improve after they scraped off the “lanh”. The perceived association between the improvement in jaundice with treatment of cranial suture diastasis or removal of “lanh” may just be a coincidental temporal relationship with the peaking of physiologic jaundice at 3–5 days before spontaneous resolution. However, for those cases that progress to pathologic hyperbilirubinemia, these traditional practices may have caused false reassurance, contribute to decreased or delayed care-seeking, and may contribute to increased risk for severe hyperbilirubinemia. The common and widespread use of traditional medicine, including herbal therapies for jaundice, has been previously reported in northern Vietnam and suggests that such therapies competed with evidence-based treatments [31]. Traditional medicine in the newborn also has been described across many cultures, sometimes causing harm, delaying care-seeking [32–35], or increasing bilirubin admission levels [32].

We found that, overall, access to care was good, and there were few reported barriers except cost. Nearly all mothers delivered at a health facility, and <1% delivered at home indicating that access to attended deliveries was not a problem. Few reported that distance, weather, and lack of transportation were potential barriers even though the primary mode of transportation is by motorcycle, and winters in northern Vietnam can be cold and rainy. Cost (17%) was the most frequently cited barrier to seeking newborn care, a finding previously reported in Vietnam [36]. A government initiative to improve access to care and decrease cost for its most vulnerable populations led to the implementation of universal, free national health insurance for children <6 years in 2005 [37]. However, this insurance remains underutilized, especially among rural populations, and the burden of out-of-pocket expenses remains high due to incomplete coverage [37–39]. Informal payments to caregivers, a prevalent practice, may add hidden costs and further deter care-seeking [40].

Maternal education may be a relatively simple and feasible approach to improve detection and increase care-seeking to prevent missed cases of hyperbilirubinemia in the community. High rates of maternal literacy and good availability of care may allow for the development of standardized education modules conducted either prenatally or post-partum. Health care providers can teach mothers about jaundice, dispel erroneous beliefs, instruct assessment of skin color under natural light, and encourage care-seeking in preference over traditional treatments. Educational campaigns should be coupled with building capacity at the district hospital for bilirubin screening and phototherapy to respond to increased demand for treatment. Investment in these interventions and equipment, however, should be done cautiously because their potential efficacy and cost-benefits are not yet known. We have no information on the actual incidence of jaundice meeting American Academy of Pediatrics criteria for phototherapy [41] or the outcome of untreated cases, and thus cannot determine if this approach will prevent many cases of kernicterus. These interventions may lead to unnecessary laboratory screening and phototherapy which may burden limited resources. Further studies to evaluate outcomes of untreated, jaundiced infants compared to those treated with phototherapy may provide the needed data to support such interventions and determine the cost-benefit of widespread implementation of phototherapy at district levels nationwide. In the interim, on-going efforts to build capacity at provincial hospitals, which care for higher risk infants, should be supported as they have been associated with decreased referrals to the NHP for both jaundice and kernicterus [29].

## Conclusions

Low maternal knowledge of jaundice, traditional beliefs and newborn care practices, and short hospital stays after birth with absence of routine newborn follow-up may prevent or delay recognition of jaundice, care-seeking, or treatment, and may account for the lower than expected frequency of phototherapy in the community. Although an education campaign to modify community care practices and traditional beliefs may improve care-seeking and providing laboratory and phototherapy capabilities at district hospitals could improve detection and treatment of hyperbilirubinemia, it is unclear whether the exchange transfusions and kernicterus cases that could be prevented would be worth the added financial burden on the healthcare system. Therefore, it would be important to obtain the data on the long-term outcomes of jaundiced infants in the community who are untreated compared to those who are treated, to determine if such costly community interventions could be justified.

## Electronic supplementary material

Additional file 1: **Hyperbilirubinemia Study Questionnaire.** Hyperbilirubinemia study questionnaire translated into English. (PDF 77 KB)

Additional file 2: **Bo cau hoi nghien cuu ve benh tang bilirubin tu do trong mau.** Hyperbilirubinemia study questionnaire in Vietnamese. (PDF 113 KB)

## Abbreviations

NHP: National Hospital of Pediatrics
HSPH: Hanoi School of Public Health.
