# Supplementary material for: Care practices and traditional beliefs related to neonatal jaundice in northern Vietnam: a population-based, cross-sectional descriptive study
Source: BMC Pediatr. 2014 Oct 14;14:264. doi: 10.1186/1471-2431-14-264 (PMC4287314; doi:10.1186/1471-2431-14-264)
Supplement: Supplementary file 1 — Additional file 1: Hyperbilirubinemia Study Questionnaire. Hyperbilirubinemia study questionnaire translated into English. (PDF 77 KB) [file 12887_2014_1189_MOESM1_ESM.pdf]

Appendix 1

Hyperbilirubinemia Study Questionnaire

Neonatal jaundice is one problem often encountered in the first 2 weeks after birth. The purpose of this study is to determine the incidence of phototherapy and risk factors contributing to neonatal jaundice in the community. This study is a collaboration between the University of California, San Francisco, and the Hanoi School of Public Health.

Inclusion Criteria: all live newborns
Exclusion Criteria: all newborns still hospitalized at 28 days of life after birth or families that cannot be contacted before 28 days after birth.
Time of interview: From 14-21 days of life. If baby is still hospitalized or family cannot be contacted prior to 21 days, then interview can be done at up to 28 days of life.

Name of interviewer: ....

Date of interview: day.............month............year.............

Interviewer ID#											
Subject ID#											

Demographic Information

1.	Mother's Name	....		
2.	Mother's Birthdate	day______ month______year_______		Western calender
3.	Baby's Name	....		
4.	Baby's sex	male<br>female	1<br>2	
5.	Baby's birthweight	Gm [ ][ ][ ][ ]<br>Don't remember	1<br>98	

Birth History

	Question	Answer		Notes
6.	Baby's date of birth	day____month_____year_____		Western calender
7.	What time was baby born?	_______h_______<br>Don't know	1<br>98	Write as 24 hour
8.	Mode of delivery	C-section<br>Vaginal<br>Forceps<br>Vacuum<br>Other (specify):......................................	1<br>2<br>3<br>4<br>99	→ # 9<br>→ #10<br>→ # 10<br>→ #10<br>→ #10

Appendix 1

Hyperbilirubinemia Study Questionnaire

9.	If C-section, then why?	………………………………………		
10.	Place of birth	Commune health station<br>District Hospital<br>Provincial Hopsital<br>Private facility<br>Home<br>Other (specify): ....…………	1<br>2<br>3<br>4<br>5<br>99	 <br><br><br>➔#13
11.	Date of Discharge	day____month_____year_____<br>Don't remember	1<br>98	Western calendar
12.	Time of Discharge	______h______<br>Don't know	1<br>98	Write as 24 hours
13.	Estimated date of delivery (by ultrasound or by estimation by health care worker)	day____month_____year_____<br>Don't remember<br>Other (specify)…………………………	1<br>98<br>99	Western Calendar
14.	Last menstrual period	day____month_____year_____<br>don't remember	1<br>98	Western Calendar
15.	How many weeks gestation did you carry the baby?	……………..weeks<br>Don't remember	1<br>98	
16.	Did baby have a cephalohematoma after birth?	Yes<br>No	1<br>2	
17a.	At time of delivery, did you have any complications?	Yes<br>No	1<br>2	 ➔ #18
17b.	If yes, what complication did you have?	………………………………………		
18.	Did baby have any complications at time of birth or while hospitalized after birth?	Yes<br>No	1<br>2	 ➔ #21
19.	If yes, what complication did baby have?	………………………………………		
20.	If baby had problems after birth, did baby receive any of the following treatments?	 antibiotics	Yes<br>1	No<br>2

Appendix 1

Hyperbilirubinemia Study Questionnaire

		IV fluids	1	2
		Oxygen supplementation	1	2
		Mechanical ventilation	1	2
		Phototherapy	1	2
		Blood exchange transfusion	1	2
		Feeds by feeding tube	1	2
		Other (specify)…………………………	99	2
21.	How many pregnancies have you had including this one?	…..………………times		
22.	How many births did you have, including this one?	………………….times		If 1 ➔ #27. If ≥ 2 ➔#23
23.	Did any of your previous children have jaundice during the first month of life?	Yes	1	
		No	2	➔ #27
		Other (specify)………………………..	99	
24.	If yes, was baby hospitalized for the jaundice?	Yes	1	
		No	2	➔ #27
		Died before hospitalization	3	➔ #27
25.	If hospitalized for jaundice, did baby receive any of the following treatments?		Yes	No
		Phototherapy	1	2
		Blood exchange transfusion	1	2
		Don't remember	1	2
		Other (specify)…………………………	98	2
26.	If hospitalized, what was the outcome of the previous baby?	Recovered, healthy, and discharged	1	
		Discharged but still having health problems	2	
		Discharged and died at home	3	
		Died in the hospital	4	
		Other (specify)…………………………	99	

Now please recall back to the baby's first week of life and answer the following questions relating to feeding and newborn care practices. (Remember this is only during the first week of life)

Feeding Practices

27.	After birth, during the first week of life, did baby get breast milk?	Yes	1	
		No	2	

Appendix 1

Hyperbilirubinemia Study Questionnaire

28.	After birth, during first week, was baby's feeding supplemented with anything else?	Yes<br>No	1<br>2	 ➔ #30
29.	If supplemented, then why?	…………………………………………..		
30.	During the first 3 days after birth, what did you feed baby?	Exclusively breast milk<br>Exclusively formula and no breast milk<br>Both breast and formula<br>Other (specify)…………………………	1<br>2<br>3<br>99	
31.	After 7 days until today, what did you feed your baby?	Exclusively breast milk<br>Both breast and formula<br>Other milk but no breast milk<br>Other (specify)… ……………………..	1<br>2<br>3<br>99	
32.	If not exclusively fed breast milk, what else did you feed baby?		Yes	No
		Baby formula	1	2
		Whole cow's milk	1	2
		Sweetened condensed milk	1	2
		Soymilk	1	2
		Rice porridge/rice water	1	2
		water	1	2
		Exclusively breastmilk	1	2
		Other (specify)………………………..	99	2
33a.	During the first 3 days after birth, on average how many times a day did you feed baby? (including breast and other milk)<br><br>(1 day means over a 24 hour period)	≤ 4 times<br>5 – 7 times<br>8-10 times<br>≥ 11 times<br>Don't remember	1<br>2<br>3<br>4<br>98	
33b.	From 4 days until 7 days after birth, on average how many times a day did you feed baby? (including breast and other milk)<br><br>(1 day means over a 24 hour period)	≤ 4 times<br>5 – 7 times<br>8-10 times<br>≥ 11 times<br>Don't know	1<br>2<br>3<br>4<br>98	
34.	Did you receive any guidance on how to breast feed?	Yes<br>No	1<br>2	 ➔ #36
35.	If yes, did you receive guidance		Yes	No

Hyperbilirubinemia Study Questionnaire

	from any of the following people?<br><br>(choose all that apply)	Physician	1	2
		Nurse/physician assistant	1	2
		Lactation consultant	1	2
		Other healthcare worker	1	2
		Mother/family member	1	2
		Other (Specify)…………………………	99	2

Newborn Care Practices

36.	During the first week after born, did you always keep baby in an enclosed room? (no sunlight, door closed)	Yes	1	
		No	2	
37.	During the first week after born, did you expose the baby to sunlight?	Yes	1	
		No	2	➔ #41
38.	How did you expose the baby to sunlight?	Open door/window, allow sunlight in but not expose to direct sunlight	1	➔ #41
		Direct exposure to sunlight	2	
		Other (specify)………………………	99	
39.	How many times did you expose baby to sunlight during the first week?	………………times	1	
		Don't know	98	
40.	How long did you exposed baby to sunlight during each exposure?	…………..minutes	1	
		Don't know	98	
41.	In your belief, during the first week after birth, what affect does taking the baby outdoors have on the baby?	Good	1	
		Harmful	2	
		No effect	3	➔# 43
		Don't know	98	➔ # 43
42.	If good or harmful, then why?	…………………………………………		
43.	In your belief, during the first week after birth, what affect does exposing the baby directly to sunlight have on the baby?	Good	1	
		Harmful	2	
		No effect	3	➔ # 45
		Don't know	98	➔ # 45
44.	If good or harmful, then why?	…………………………………………		
45.	During the first week after birth, did you use any traditional remedies, herbal medicines, or Chinese medicines to keep baby	Yes	1	
		No	2	➔ #47

Appendix 1

Hyperbilirubinemia Study Questionnaire

	healthy and prevent illness?			
46.	If yes, what did you use?<br>…………………………………………………………………………………………			
47.	During the first week after birth, did baby have any health problems?	Yes<br>No	1<br>2	 ➔ #51a
48.	If yes, what problem did baby have?	………………………………………………………………		
49.	Did you use any traditional remedies, herbal medicines, or Chinese medicines to treat baby at home before taking the baby to a doctor?	Yes<br>No	1<br>2	 ➔# 51a
50.	If yes, what did you use?<br>...			
51a.	Do you put moth balls in your clothes drawer?	Yes<br>No	1<br>2	
51aa.	Do you ever use mentholated oils on baby's skin or on baby's clothes?	Yes<br>No	1<br>2	
51b.	Other than cotton, alcohol, and tape, do you use any traditional practices or medicines to care for baby's umbilicus?	Yes<br>No	1<br>2	 ➔ #52
51c.	If yes, specify	…………………………………..		

Jaundice

52.	Prior to baby's birth, did you ever hear about neonatal jaundice?	Yes<br>No	1<br>2	 ➔ # 56
53.	If yes, where did you hear it from?<br><br>(Choose all that apply)		Yes	No
		Elders/family members/acquaintances	1	2
		T.V./newsprint/books/self-learned	1	2
		Physicians/nurses/health care workers	1	2
		Previous baby was jaundiced	1	2
		Other (specify)………………………	99	2
54.	Prior to baby's birth, did you think that neonatal jaundice can be harmful?	Yes<br>No<br>Don't know	1<br>2<br>98	 ➔ # 56<br>➔ # 56
55.	If yes, where did you hear this from?<br><br>(choose all that apply)		Yes	No
		Elders/family members/acquaintances	1	2
		T.V./newsprint/books/self-learned	1	2
		Physicians/nurses/health care workers	1	2

Appendix 1

Hyperbilirubinemia Study Questionnaire

		Previous baby was jaundiced	1	2
		Heard of cases where babies needed phototherapy, blood exchange transfusion, or died from jaundice	1	2
		Other (specify)………………………	99	2
56.	After birth, did you receive any guidance on monitoring neonatal jaundice?	Yes<br>No	1<br>2	➔ # 58
57.	If yes, who did you receive guidance from?<br><br>(Choose all that apply)		Yes	No
		Elders/family members/acquaintances	1	2
		T.V./newsprint/books/self-learned	1	2
		Physicians/nurses/health care workers	1	2
		I know because previous child jaundiced	1	2
		Khác (ghi rõ)………………………..	99	2
58.	Did baby become jaundice during the first week after birth?	Yes<br>No	1<br>2	 ➔ #63
59.	When did baby first appeared jaundiced?	Day___Month____Year______<br>Don't remember	1<br>98	Western calendar
60.	Did you worry when you saw that the baby was jaundiced?	Yes<br>No	1<br>2	 ➔ # 63
61.	If you were worried, did you take baby to a health care facility to be checked?	Yes<br>No	1<br>2	➔ # 63
62.	If no, then why?	………………………………………………………		

Healthcare Practices

The following questions relate to events occurring 2 weeks after birth.

63.	During the first 2 weeks afgter birth, did you take baby for a well baby check-up visit? (Baby was not sick.)	Yes<br>No	1<br>2	 ➔ # 65
64.	If yes, when was the first time did you take baby to be seen?	Day_____ Month______Year______<br>Don't know	1<br>98	Western calendar
65.	During the first 2 weeks after birth, did baby have any of the following problems?<br>If YES to ANY of questions 65a-65h, continue to question #66.			

Appendix 1

Hyperbilirubinemia Study Questionnaire

	If NO to ALL questions 65a-65h, then skip to question #68.		Yes	No
65a.		Jaundice	1	2
65b.		Poor feeding/stopped feeding	1	2
65c.		Breathing problems	1	2
65d.		Sleeping a lot, lethargic, difficult to arouse	1	2
65e.		Poor voiding or no stooling	1	2
65f.		Seizures	1	2
65g.		Fever/infection	1	2
65h.		Other (specify)…………………………………………..	99	2
66.	Did you take baby to be checked for any of those problems?	Yes<br>No	1<br>2	 ➔ #68
67.	When did you take baby to be checked?	day_____ month______year______<br>Don't remember	1<br>98	Western calendar
68.	Was the baby hospitalized for any reason? (Meaning after baby was discharged home, had heath problems, and readmitted)	Yes<br>No	1<br>2	 ➔ # 78

Questions Regarding Rehospitalization

69.	Where did you take the baby to be hospitalized?	Chí Linh District Hospital<br>Hải Dương Provincial Hospital<br>Quảng Ninh Swedish Hospital<br>National Hospital of Pediatrics<br>Other (specify)………………	1<br>2<br>3<br>4<br>99	
70.	What was the main reason for baby's hospitalization?	………………………………………		
71.	When was baby admitted?	day____ month_____ year____<br>Don't remember	1<br>98	
72.	If baby was examined/hospitalized at district of provincial hospital, was baby subsequently transferred to the National Hospital of Pediatrics?	Yes<br>NO<br>Brought directly to the NHP	1<br>2<br>3	 ➔ #74<br>➔ # 74
73.	If transferred to the NHP, what day did that occur?	day____ month_____ year____<br>Don't remember	1<br>98	
74.	How many days did baby stay in the hospital? (total time in all	………………days		

Hyperbilirubinemia Study Questionnaire

	hospitalized)			
75.	During the time that baby was hospitalized, did baby receive any of the following treatments?		Yes	No
75a.		Phototherapy	1	2
75b.		Blood exchange transfusion	1	2
75c.		IV fluids	1	2
75d.		Oxygen supplementation	1	2
75e.		Mechanical ventilation/bag mask ventilation	1	2
75e.		Antibiotics	1	2
75f.		Tube feedings	1	2
75g.		Other (specify)……………………………..………	99	2
76.	What was the physician's diagnosis of baby's problem?	………………………………………	1	
		Don't know	98	
77.	Outcome after discharged from hospital:	Recovered, healthy, discharged home	1	
		Recovered, discharged, but still having residual health problems	2	
		Discharged home and died	3	
		Died at hospital	4	
		Other (specify)………………………	99	
78.	Hypothetically, if you needed to take baby to be seen at a health care facility, do any of the following make it difficult for you to seek care?		Yes	NO
78a.		Cost	1	2
78b.		Distance from home to health care facility too far	1	2
78c.		No mode of transportation	1	2
78d.		Bad impression of health care workers from previous experience	1	2
78e.		No time to take baby or unable to get time off from work	1	2
78f.		Baby too small, don't want to take baby outside of the home	1	2
78g.		Bad weather/too cold/rain	1	2
78h.		Other (specify)……………………...……………………	99	2

Thank you for participating!
