# Supplementary material for: Care practices and traditional beliefs related to neonatal jaundice in northern Vietnam: a population-based, cross-sectional descriptive study
Source: BMC Pediatr. 2014 Oct 14;14:264. doi: 10.1186/1471-2431-14-264 (PMC4287314; doi:10.1186/1471-2431-14-264)
Supplement: Supplementary file 2 — Additional file 2: Bo cau hoi nghien cuu ve benh tang bilirubin tu do trong mau. Hyperbilirubinemia study questionnaire in Vietnamese. (PDF 113 KB) [file 12887_2014_1189_MOESM2_ESM.pdf]

Appendix 2

Bộ câu hỏi nghiên cứu về bệnh tăng bilirubin tự do trong máu

Vàng da sơ sinh là một vấn đề thường gặp trong 2 tuần đầu tiên sau khi sinh. Mục đích của nghiên cứu này nhằm xác định tỉ lệ hiện mắc và các yếu tố nguy cơ gây ra vàng da ở trẻ sơ sinh trong cộng đồng. Nghiên cứu được hợp tác giữa trường Đại Học Y California tại San Francisco và Đại Học Y Tế Công Cộng Hà Nội.

Đối tượng đích của nghiên cứu: bao gồm tất cả các trẻ sơ được sinh ra sống.

Ngoại trừ (không phỏng vấn):

- những trẻ phải ở lại bệnh viên/cơ sở y tế 28 ngày trở lên sau khi sinh mới được về nhà hay những gia đình không liên lạc được trước 28 ngày tuổi

Thời điểm phỏng vấn: Từ 14-21 ngày tuổi. Nếu trẻ vẫn còng nằm tại bệnh viện hây không liên lạc gia đình được trước 21 ngày, thì có thể phỏng vấn đến 28 ngày tuổi.

Họ và tên điều tra viên: ....

Ngày phỏng vấn: ngày............tháng...........năm............

Mã hộ phỏng vấn: | | | | | | | | | |

Mã cá nhân: | | | | | | | | | | | |

Thông tin về nhân khẩu học

1.	Họ và tên mẹ	....		Ghi chú
2.	Ngày tháng năm sinh của mẹ	ngày______ tháng______năm_______		Dương lịch
3.	Họ và tên trẻ	....		
4.	Giới tính của trẻ	Nam<br>Nữ	1<br>2	
5.	Cân nặng của trẻ khi sinh	Gam ☐☐☐☐<br>Không nhớ	1<br>98	

Các thông tin liên quan đến việc sinh đẻ

Stt	Câu hỏi	Phương án trả lời		Ghi Chú
6.	Ngày sinh của trẻ:	ngày____tháng_____năm_____		Dương lịch
7.	Cháu sinh ra lúc mấy giờ?	_______h_______<br>Không nhớ	1<br>98	Ghi theo 24 giờ
8.	Phương pháp sinh	Mổ đẻ	1	➔ câu 9
		Đẻ thường (không có sự can thiệp của y tế)	2	➔ câu 10
		Đẻ có sự can thiệp của y tế (kẹp fooc-xép)	3	➔ câu 10
		Đẻ có sự can thiệp của y tế (sử dụng giác hút)	4	➔ câu 10
		Khác (ghi rõ):..	99	➔ câu 10

Appendix 2

Bộ câu hỏi nghiên cứu về bệnh tăng bilirubin tự do trong máu

9.	Nếu có mổ đẻ, xin chị cho biết tại sao phải mổ?	…………………………………………		
10.	Nơi sinh	Trạm y tế xã<br>Bệnh viện huyện<br>Bệnh viện tỉnh<br>Cơ sở y tế tư nhân<br>Tại nhà<br>Địa điểm khác (ghi rõ): ....…………	1<br>2<br>3<br>4<br>5<br>99	 <br><br><br>➔câu 13
11.	Ngày xuất viện	ngày____tháng_____năm_____<br>Không nhớ	1<br>98	Dương lịch<br>**Nếu phải ở lại BV 5 ngày trở lên thì dừng PV**
12.	Giờ xuất viện	______h______<br>Không nhớ	1<br>98	Ghi theo 24 giờ
13.	Ngày sinh con theo dự kiến (theo siêu âm hay chuẩn đoán của CBYT)	ngày____tháng_____năm_____<br>Không nhớ<br>Khác (ghi rõ)………………………	1<br>98<br>99	Dương lịch
14.	Ngày kinh cuối cùng:	ngày____tháng_____năm_____<br>Không nhớ	1<br>98	Dương lịch
15.	Chị mang thai cháu được bao nhiêu tuần thì sinh?	……………..Tuần<br>Không nhớ	1<br>98	**Nếu ≤34 tuần thai thì dừng PV**
16.	Cháu có bị u/tụ máu trên đầu sau khi sinh không?	Có<br>Không	1<br>2	
17a.	Trong và sau khi sinh, chị có gặp vấn đề gì không?	Có<br>Không	1<br>2	 ➔ câu 18
17b.	Nếu có, xin chị cho biết vấn đề đó là gì?	………………………………………		
18.	Cháu bé có gặp vấn đề gì khi chị đẻ hoặc trong thời gian nằm trong viện sau khi sinh không?	Có<br>Không	1<br>2	 ➔ câu 21

Appendix 2

Bộ câu hỏi nghiên cứu về bệnh tăng bilirubin tự do trong máu

19.	Nếu **có**, xin chị cho biết cháu bị vấn đề gì?	………………………………………		
20.	**Nếu có vấn đề** sau khi sinh**,** mong chị có thể cho biết cháu đã được chữa trị theo những cách nào dưới đây		Có	Không
		Kháng sinh	1	2
		Truyền dịch	1	2
		Thở bằng oxy	1	2
		Gắn máy thở	1	2
		Chiếu đèn	1	2
		Thay máu	1	2
		Cho ăn bằng ống thông	1	2
		Khác (ghi rõ)………………………….	99	2
21.	Chị đã mang thai bao nhiêu lần, kể cả lần mang thai cháu bé này?	…..………………lần		
22.	Chị đã sinh bao nhiêu lần, kể cả lần sinh này?	………………….lần		Nếu 1 lần ➔ câu 27. Nếu ≥ 2 lần➔câu 23
23.	Những đứa con trước của chị có cháu nào bị vàng da trong tháng tuổi đầu tiên không?	Có	1	
		Không	2	➔ câu 27
		Khác (ghi rõ)…………………………..	99	
24.	Nếu có, cháu có phải nhập viện vì bị vàng da không?	Có	1	
		Không	2	➔ câu 27
		Tử vong trước khi nhập viện	3	➔ câu 27
25.	Nếu có nhập viện, cháu đó được điều trị bằng phương pháp nào?		Có	Không
		Chiếu đèn	1	2
		Thay máu	1	2
		Không nhớ	1	2
		Khác (gi rõ)…………………………	98	2
26.	Nếu có nhập viện, xin chị cho biết kết quả của đứa con trước?	Qua khỏi, bình phục, và được xuất viện	1	
		Xuất viện nhưng vẫn có vấn đề về sức khỏe	2	
		Xuất viện về nhà thì mất	3	
		Mất tại bệnh viện	4	
		Khác (ghi rõ)…………………………	99	

Appendix 2

Bộ câu hỏi nghiên cứu về bệnh tăng bilirubin tự do trong máu

Bây giờ mong chị hồi tưởng lại tuần đầu tiên khi cháu bé chào đời và trả lời các câu hỏi có liên quan tới việc thực hành và vấn đề chăm sóc trẻ dưới đây **(chú ý chỉ tuần đầu tiên sau khi trẻ chào đời).**

Thực hành cho trẻ ăn

27.	Sau khi sinh, **trong tuần tuổi đầu tiên,** cháu có được bú sữa mẹ không?	Có<br>Không	1<br>2	
28.	Sau khi sinh, **trong tuần tuổi đầu tiên,** cháu có được bú thêm/ăn thêm sữa ngoài không?	Có<br>Không	1<br>2	 ➔ câu 30
29.	Nếu có bú/ăn sữa ngoài, xin chị cho biết tại sao?	…………………………………………..		
30.	Trong **3 ngày đầu tiên** sau khi sinh, chị cho cháu bú sữa loại nào?	Hoàn toàn sữa mẹ, và không cho thêm sữa ngoài<br>Hoàn toàn sữa ngoài, và không bú sữa mẹ<br>Cả hai sữa mẹ và sữa ngoài<br>Khác (ghi rõ)……………………………	1<br>2<br>3<br>99	
31.	**Sau 7 ngày đầu tiên cho đến hôm nay**, chị cho cháu bú sữa loại nào?	Hoàn toàn sữa mẹ, và không cho thêm sữa ngoài<br>Cả hai sữa mẹ và sữa ngoài<br>Không bú sữa mẹ<br>Khác (ghi rõ)……………………………	1<br>2<br>3<br>99	

			Có	Không
32.	Nếu bú ngoài sữa mẹ, chị cho cháu bú/ăn gì thêm?	Sữa nhân tạo dành cho trẻ em	1	2
		Sữa tươi	1	2
		Sữa đặc có đường	1	2
		Sữa đậu nành	1	2
		Nước cháo/nước cơm	1	2
		Nước	1	2
		Cháu chỉ bú sữa mẹ hoàn toàn từ sinh đến bây giờ	1	2
		Khác (Ghi rõ)…………………………..	99	2

33a.	**Trong 3 ngày đầu sau khi sinh**, trung bình 1 ngày chị cho cháu bú mấy lần? (Kể cả sữa mẹ và sữa ngoài)<br><br>(**1 ngày nghĩa là cả ngày và qua đêm, trong 24 giờ**)	≤ 4 lần<br>5 – 7 lần<br>8-10 lần<br>≥ 11 lần<br>Không nhớ	1<br>2<br>3<br>4<br>98	

Appendix 2

Bộ câu hỏi nghiên cứu về bệnh tăng bilirubin tự do trong máu

33b.	**<u>Từ ngày thứ 4 đến ngày thứ 7 sau khi sinh,</u>** trung bình 1 ngày chị cho cháu bú mấy lần? (Kể cả sữa mẹ và sữa ngoài)<br><br>(**1 ngày nghĩa là cả ngày và qua đêm, trong 24 giờ**)	≤ 4 lần<br>5 – 7 lần<br>8-10 lần<br>≥ 11 lần<br>Không nhớ	1<br>2<br>3<br>4<br>98	
34.	Chị có nhận được bất cứ sự chỉ dẫn nào về cách cho con bú không?	Có<br>Không	1<br>2	 ➔ câu 36

			Có	Không
35.	Nếu có, mong chị cho biết chị được hướng dẫn từ đâu?<br><br>(**Câu hỏi nhiều lựa chọn**)	Bác sĩ	1	2
		Y tá/Y sĩ	1	2
		Chuyên gia về nuôi con bằng sữa mẹ	1	2
		Người cung cấp dịch vụ CSSK khác	1	2
		Mẹ/thành viên trong gia đình	1	2
		Khác (Ghi rõ)…………………………	99	2

<u>Các câu hỏi liên quan đến thực hành chăm sóc trẻ</u>

36.	**<u>Trong tuần đầu sau khi sinh</u>**, chị có luôn luôn giữ cháu trong phòng kín không? (không có ánh sáng mặt trời, cửa sổ luôn đóng)	Có<br>Không	1<br>2	
37.	**<u>Trong tuần đầu</u>** chị có cho cháu tắm nắng không?	Có<br>Không	1<br>2	 ➔ câu 41
38.	Chị cho cháu tắm nắng bằng cách nào?	Mở cửa/cửa sổ cho ánh sáng vào phòng nhưng không cho cháu tiếp xúc trực tiếp với ánh nắng mặt trời<br>Cho cháu tiếp xúc trực tiếp với ánh nắng mặt trời<br>Khác (ghi rõ)…………………………	1<br>2<br>99	➔ câu 41
39.	Chị cho cháu tắm nắng bao nhiêu lần trong tuần đầu tiên?	………………lần<br>Không nhớ	1<br>98	
40.	Mỗi lần tắm nắng kéo dài khoảng bao lâu?	…………..phút<br>Không biết	1<br>98	
41.	Theo chị, **<u>trong tuần đầu</u>**, cho trẻ ra ngoài trời sẽ tác động tới trẻ như thế nào?	Tốt<br>Không tốt<br>Không có tác động gì hết<br>Không biết	1<br>2<br>3<br>98	 <br>➔ câu 43<br>➔ câu 43
42.	Nếu tốt hay không tốt, tại sao?	………………………………………		

Appendix 2

Bộ câu hỏi nghiên cứu về bệnh tăng bilirubin tự do trong máu

43.	Theo chi, **trong tuần đầu**, cho trẻ tiếp xúc trực tiếp với ánh nắng mặt trời sẽ có tác động như thế nào?	Tốt<br>Không tốt<br>Không có tác động gì hết<br>Không biết	1<br>2<br>3<br>98	 <br>➔ câu 45<br>➔ câu 45
44.	Nếu tốt hay không tốt, tại sao?	…………………………………………		
45.	**Trong tuần đầu**, chị có dùng phương pháp dân gian, thuốc nam, hay đông y nào để giữ cho cháu khỏe và phòng bệnh cho cháu không?	Có<br>Không	1<br>2	 ➔ câu 47
46.	Nếu có thì ghi rõ đó là phương pháp gì? (ghi chi tiết phương pháp đó ra): ……………………………………………………………………………………………………			
47.	**Trong tuần tuổi đầu**, cháu bé có gặp bất cứ vấn đề gì về sức khỏe không?	Có<br>Không	1<br>2	 ➔ câu 51a
48.	Nếu có, cháu gặp vấn đề gì?	…………………………………………………………………		
49.	Chị có sử dụng bất kì phương pháp dân gian, thuốc nam, đông y hay tây y để chữa trị cho cháu **tại nhà** trước khi khám bác sĩ không?	Có<br>Không	1<br>2	 ➔ câu 51a
50.	Nếu có thì ghi rõ đó là phương pháp gì? (ghi chi tiết phương pháp đó ra): ………………………………………………………………………………………………………			
51a.	Ở nhà chị có bỏ băng phiến vào trong tủ quần áo không?	Có<br>Không	1<br>2	
51aa.	Chị có bao giò sức dầu (trắng, xanh, đỏ, vv) tren cháu hoạc quàn áo cháu không?	Có<br>Không	1<br>2	
51b.	Ngoài cồn, bông, băng, gia đình mình có dùng phương pháp dân gian hoặc thuốc gì khác khi băng rốn cho cháu không?	Có<br>Không	1<br>2	 ➔ câu 52
51c.	Nếu có, ghi rõ:	…………………………………..		

Bệnh vàng da

52.	**Trước khi** chị sinh cháu bé này, chị đã từng nghe nói về vàng da sơ sinh không?	Có<br>Không	1<br>2	 ➔ câu 56
53.	Nếu có, chị nghe về vàng da sơ sinh từ đâu?<br><br>**(Câu hỏi nhiều lựa chọn)**		Có	Không
		Các cụ nói/gia đình/người quen	1	2
		Qua ti vi/sách báo/tự tìm hiểu	1	2
		Bác sĩ/Y tá/CBYT giải thích	1	2
		Con trước bị vàng da	1	2
		Khác (ghi rõ)…………………………	99	2

Appendix 2

Bộ câu hỏi nghiên cứu về bệnh tăng bilirubin tự do trong máu

54.	**<u>Trước khi</u>** chị sinh cháu bé này, chị có nghĩ rằng vàng da sơ sinh có thể gặp nguy hiểm không?	Có<br>Không<br>Không biết	1<br>2<br>98	 ➔ câu 56<br>➔ câu 56
55.	Nếu có, chị cho biết chị đã nghe từ đâu?<br><br>**(Câu hỏi nhiều lựa chọn)**		Có	Không
		Các cụ nói/gia đình/người quen	1	2
		Tự tìm hiểu/nghe qua ti vi hoặc sách báo	1	2
		Bác sĩ/Y tá/CBYT giải thích	1	2
		Tôi biết tại vì con trước bị vàng da	1	2
		Có nghe đến trường hợp trẻ sơ sinh khác phải chiếu đèn, thay máu, hoặc tử vọng do bệnh vàng da	1	2
		Khác (ghi rõ)…………………………	99	2
56.	Sau khi sinh, chị có được hướng dẫn cách theo dõi vàng da sơ sinh không?	Có<br>Không	1<br>2	➔ câu 58
57.	Nếu có, chị cho biết chị đã được hướng dẫn từ đâu?<br><br>**(Câu hỏi nhiều lựa chọn)**		Có	Không
		Các cụ nói/gia đình/người quen	1	2
		Tự tìm hiểu/nghe qua ti vi hoặc sách báo	1	2
		Bác sĩ/Y tá/CBYT giải thích	1	2
		Tôi biết tại vì con trước bị vàng da	1	2
		Khác (ghi rõ)………………………..	99	2
58.	Cháu có bị vàng da **<u>trong tuần đầu</u>** sau khi sinh không?	Có<br>Không	1<br>2	 ➔ câu 63
59.	Khi chị phát hiện da cháu có màu vàng, lúc đó là ngày nào?	Ngày___Tháng____Năm______<br>Không nhớ	1<br>98	Dương lịch
60.	Chị có lo lắng khi biết da cháu có màu vàng không?	Có<br>Không	1<br>2	 ➔ câu 63
61.	Nếu có lo lắng, chị có đem cháu đi tới cơ sở y tế để khám vàng da không?	Có<br>Không	1<br>2	➔ câu 63
62.	Nếu không, tại sao?	………………………………………………………		

Appendix 2

Bộ câu hỏi nghiên cứu về bệnh tăng bilirubin tự do trong máu

Chăm sóc sau sinh

Những câu hỏi tiếp theo sẽ liên quan tới những sự kiện xảy ra trong suốt **2 tuần đầu** sau khi sinh của trẻ.

63.	**Trong 2 tuần đầu sau khi sinh**, chị có cho cháu đi kiểm tra sức khỏe không? (Nghĩa là không có vấn đề gì, nhưng vẫn đến để khám sức khỏe)	Có<br>Không	1<br>2	 ➔ câu 65
64.	Nếu có, **lần đầu tiên** chị đem cháu đi khám là ngày nào?	ngày_____ tháng______năm______<br>Không nhớ	1<br>98	Dương lịch

65.	**Trong 2 tuần đầu sau khi sinh**, cháu có bị những vấn đề sau đây không?<br>**Nếu trả lời Có trong bất cứ câu hỏi nào 65a-65h, trả lời tiếp câu hỏi 66.**<br>**Nếu trả lời Không trong tất cả câu hỏi 65a-65h, chuyển sang 68.**	Có	Không
65a.	Vàng da	1	2
65b.	Ăn ít đi/Bỏ bú	1	2
65c.	Có vấn đề về thở	1	2
65d.	Ngủ nhiều, li bì, khó đánh thức	1	2
65e.	Đi tiểu ít hoặc không đi đại tiện	1	2
65f.	Co giật	1	2
65g.	Sốt/nhiễm trùng	1	2
65h.	Khác (ghi rõ)…………………………………………..	99	2

66.	Chị có đem cháu đi khám do những vấn đề đó không?	Có<br>Không	1<br>2	 ➔ câu 68
67.	Chị cho cháu đi khám ngày nào?	ngày_____ tháng______năm______<br>Không nhớ	1<br>98	Dương lịch
68.	Cháu có phải nhập viện lại do những vấn đề nào không?<br>(Nghĩa là cháu đã về nhà sau khi sinh nhưng do có vấn đề về sức khỏe nên phải nhập viện lại)	Có<br>Không	1<br>2	 ➔ câu 78

Câu hỏi liên quan đến việc nhập viện của trẻ

69.	Chị cho cháu tới bệnh viện nào để khám?	Bệnh viện đa khoa Chí Linh<br>Bệnh viện tỉnh Hải Dương<br>Bệnh viện Thụy Điển Quảng Ninh<br>Bệnh viện Nhi Trung Ương<br>Cơ sở y tế khác (Ghi rõ)………………	1<br>2<br>3<br>4<br>99	
70.	Lí do cháu được nhập vào viện? (Nguyên nhân chính)	……………………………………………		
71.	Cháu được nhập viện vào ngày	ngày____ tháng_____ năm____	1	

Appendix 2

Bộ câu hỏi nghiên cứu về bệnh tăng bilirubin tự do trong máu

	nào?	Không nhớ	98	
72.	Nếu cháu có được khám/nhập tại BV huyện/ tỉnh, sau đó cháu có được chuyển đến BV Nhi Trung Ương không?	Có<br>Không<br>Chuyển đến BV Nhi Trung Ương từ đầu	1<br>2<br>3	 ➔ câu 74<br>➔ câu 74
73.	Nếu có chuyển đến BV Nhi Trung Ương, thì chuyển ngày nào?	ngày____ tháng_____ năm____<br>Không nhớ	1<br>98	
74.	Cháu nằm viện bao lâu? (Tổng thời gian tất cả BV)	………………ngày		

75.	Trong thời gian cháu ở viện có được nhận bất cứ cách điều trị nào dưới đây không?	Có	Không
75a.	Chiếu đèn	1	2
75b.	Thay máu	1	2
75c.	Truyền dịch	1	2
75d.	Thở oxy	1	2
75e.	Hỗ trợ thở bằng máy/bóp bóng	1	2
75e.	Kháng sinh	1	2
75f.	Cho ăn bằng ống thông	1	2
75g.	Khác (ghi rõ) ……………………………...………	99	2

76.	Bác sĩ chẩn đoán cháu bị làm sao?	……………………………………………<br>Không biết	1<br>98	
77.	Kết quả sau khi xuất viện:	Qua khỏi, bình phục, và được xuất viện<br>Qua khỏi và được xuất viện nhưng vẫn có vấn đề về sức khỏe<br>Xuất viện về nhà thì mất<br>Mất tại bệnh viện<br>Khác (ghi rõ)…………………………	1<br>2<br>3<br>4<br>99	

78.	Giả sử chị cần phải đưa trẻ đi khám bệnh thì chị có gặp khó khăn gì sau đây không?	Có	Không
78a.	Chi phí	1	2
78b.	Từ nhà tới cơ sở y tế quá xa	1	2
78c.	Không có phương tiện để đưa cháu đi	1	2
78d.	Có ấn tượng không tốt về CBYT chăm sóc sức khỏe trước đây	1	2
78e.	Không có thời gian để đưa con đi hoặc không xin nghỉ làm được	1	2
78f.	Cháu nhỏ quá, không muốn đem cháu ra ngoài nhà	1	2
78g.	Thời tiết không tốt/lạnh quá/mưa	1	2
78h.	Khác (Ghi rõ)……………………….............………………	99	2

Cám ơn chị đã tham gia phỏng vấn!
